# The perceptions of healthcare practitioners on obesity management in Peninsular Malaysia: a cross-sectional survey

**DOI:** 10.1186/s12913-023-09759-z

**Published:** 2023-07-10

**Authors:** Nor Akma Yunus, Grant Russell, Rosediani Muhamad, Sze-Ee Soh, Elizabeth Sturgiss

**Affiliations:** 1 School of Primary and Allied Health Care, Monash University Peninsula Campus, Frankston, Victoria 3199 Australia; 2 Department of Family Medicine, School of Medical Sciences, Universiti Sains Malaysia Health Campus, Kubang Kerian, Kelantan Malaysia; 3 Department of General Practice, School of Public Health and Preventive Medicine, Monash University, Melbourne, Victoria Australia; 4 Department of Physiotherapy, School of Primary and Allied Health Care, Monash University, Frankston, Victoria Australia; 5 Department of Epidemiology and Preventive Medicine, School of Public Health and Preventive Medicine, Monash University, Melbourne, Victoria Australia

**Keywords:** Obesity management, Practitioners, Perspectives, Weight stigma, Healthcare, Asia

## Abstract

**Background:**

Practitioners’ perceptions of patients with obesity and obesity management shape their engagement in obesity care delivery. This study aims to describe practitioners’ perceptions, experiences and needs in managing patients with obesity, determine the extent of weight stigma among health practitioners, and identify the factors associated with negative judgment towards patients with obesity.

**Methods:**

A cross-sectional online survey was conducted from May to August 2022 with health practitioners commonly involved in obesity management in Peninsular Malaysia, including doctors in primary care, internal medicine and bariatric surgery, and allied health practitioners. The survey explored practitioners’ perceptions, barriers and needs in managing obesity, and evaluated weight stigma using the Universal Measures of Bias – Fat (UMB Fat) questionnaire. Multiple linear regression analysis was used to identify demographic and clinical-related factors associated with higher negative judgment towards patients with obesity.

**Results:**

A total of 209 participants completed the survey (completion rate of 55.4%). The majority (n = 196, 94.3%) agreed that obesity is a chronic disease, perceived a responsibility to provide care (n = 176, 84.2%) and were motivated to help patients to lose weight (n = 160, 76.6%). However, only 22% (n = 46) thought their patients were motivated to lose weight. The most frequently reported barriers to obesity discussions were short consultation time, patients’ lack of motivation, and having other, more important, concerns to discuss. Practitioners needed support with access to multi-disciplinary care, advanced obesity training, financing, comprehensive obesity management guidelines and access to obesity medications. The mean (SD) of the UMB Fat summary score was 2.99 (0.87), with the mean (SD) domain scores ranging between 2.21 and 4.36 (1.06 to 1.45). No demographic and clinical-related factors were significantly associated with negative judgment from the multiple linear regression analyses.

**Conclusion:**

Practitioners in this study considered obesity a chronic disease. While they had the motivation and capacity to engage in obesity management, physical and social opportunities were the reasons for not discussing obesity with their patients. Practitioners needed more support to enhance their capability and opportunity to engage with obesity management. Weight stigma in healthcare settings in Malaysia should be addressed, given the possibility of hindering weight discussions with patients.

**Supplementary Information:**

The online version contains supplementary material available at 10.1186/s12913-023-09759-z.

## Background

There is a pressing need to address obesity at all levels of the healthcare system as the prevalence of obesity increases globally [1]. International guidelines recommend that healthcare practitioners diagnose the condition, screen for obesity-related comorbidities and complications, discuss obesity treatment options, refer patients for multi-disciplinary team care, and provide health education regarding obesity and its implications on health and well-being [2–5]. While healthcare practitioners generally agree that obesity is a chronic disease [6] and recognise their responsibilities in supporting patients with obesity [6, 7], obesity management is considered challenging [8].

Practitioners’ engagement in obesity management can be explored through the lens of the Capability, Opportunity, Motivation and Behaviour (COM-B) model, a behavioural analysis framework that serves as the central component of the Behaviour Change Wheel, a comprehensive theoretical framework for behaviour change intervention [9]. The COM-B model proposes that people’s engagement in a behaviour requires motivation, opportunity and capability, where opportunity and capability operate as gatekeepers that affect motivation for the behaviour [9]. Literature shows that practitioners acknowledge the serious health implications of obesity [7, 10, 11] and their responsibilities in obesity management [6, 7, 10], which can motivate them engage in obesity management [9]. However, they reported limitations in capability, such as inadequate training in obesity management [8, 12, 13] to meet the demand and answer questions from patients [8]. Limited opportunities such as short consultation time [7, 10], limited financial and human resources [7], and inadequate access to multi-disciplinary approaches [7, 8] are cited by healthcare practitioners as barriers to obesity management.

Healthcare practitioners commonly report a patient’s lack of interest or motivation to lose weight as a challenge in obesity management [6, 7, 14]. Blaming the patients’ lack of motivation for unsuccessful weight management implies that practitioners believe that obesity is mostly attributed to the patient, as suggested by Attribution theory [15]. Attribution theory is concerned with who and what we attribute the causality of an event to, as well as the emotional and motivational consequences of these beliefs [15]. If a condition such as obesity is attributed by others to a factor within the individual’s control, this could contribute to personal blame, promote a lack of compassion and reduce the offers of help [15]. People with obesity are often negatively judged by others because of their body size, with stereotypical behaviours such as laziness and lack of self-discipline [16]. This negative judgment leads to poor treatment and discrimination against people with obesity [17, 18]. Moreover, patients with obesity have expressed their need for guidance and support from their healthcare practitioners on their weight loss journey in a non-judgemental way [19–22].

Additionally, if practitioners have negative perceptions of patients with obesity, while also attributing obesity to personal responsibility, this further contributes to weight stigma in healthcare [23, 24]. Weight stigma in healthcare settings has been shown to affect healthcare quality and clinical decision-making [25]. Patients who experience weight stigma tend to limit their interactions with the healthcare system [26], which hinders their efforts to tackle obesity [25, 27, 28]. Some practitioners were less inclined to perform cervical cancer screening on patients with obesity, which led to delays in having the screening [29]. Practitioners also spent less time on health education for patients with obesity [30, 31]. Moreover, individuals who experienced weight stigma showed increased eating behaviours, decreased self-regulation and increased cortisol levels, likely contributing to weight gain and poor health outcomes for individuals [32]. Numerous factors have been found to be associated with high weight stigma in healthcare practitioners. Practitioners personal factors include negative attitudes towards people with obesity [33, 34], younger and older age [34], male gender [34], high and low BMI status [34], and previous success in weight loss [34]. The reported clinical-related factors were lack of competence in obesity management [34], unfavourable contact time with patients [34], more and less professional experience [34], poor role modelling [34] and lack of resources [34].

Malaysia has the highest prevalence of obesity in the southeast Asian region [35]. Despite the high prevalence, health practitioners’ perceptions of patients with obesity and obesity management are infrequently explored in the local studies [36]. A survey among primary care doctors on the east coast of Malaysia revealed that they have a moderate level of self-efficacy in obesity counselling [37]. In this study, having good knowledge of obesity management guidelines and being involved in a non-communicable disease team was associated with higher self-efficacy [37]. In another local study, community pharmacists expressed their willingness to help patients with their weight loss attempts [38]. However, the participants’ weight management practices were limited to screening, blood pressure and blood sugar measurement, giving dietary and exercise advice and selling weight loss products [38]. The community pharmacists also mentioned a lack of staff and a perceived lack of patient willingness to utilise weight management services within the pharmacy as the barriers to obesity care [38]. To date, there have been no studies in Malaysia that explore weight stigma and negative judgment towards people with obesity among healthcare practitioners. Given the limited research on the perspectives of healthcare practitioners in the Malaysian context, the aim of this study was to explore the healthcare practitioners’ perspectives on obesity management in Peninsular Malaysia. Our specific objectives were to:

1. describe healthcare practitioners’ perceptions, experience and needs in managing patients with obesity in Peninsular Malaysia.

2. determine the extent of weight stigma among healthcare practitioners in Peninsular Malaysia.

3. identify the sociodemographic and clinical-related factors associated with negative judgment towards patients with obesity among healthcare practitioners in Peninsular Malaysia.

## Methods

This study was an online cross-sectional survey conducted from 23 May 2022 to 29 August 2022. The protocol was approved by the Monash University Human Research Ethics Committee (project ID 28,431) and the Universiti Sains Malaysia Human Research Ethics Committee (USM/JEPeM/21,100,673). The reporting was guided by the Strengthening the reporting of observational studies in epidemiology (STROBE) statement [39].

### Setting and participants

Obesity healthcare services in Peninsular Malaysia are delivered in some primary care clinics and hospitals in the public and private sectors through clinical consultations and management or weight loss programs [Authors, 2022, under review] [40]. However, each service’s availability, structure and methods vary according to the preferences of the managing teams and the feasibility of human resources [40]. Our survey involved healthcare practitioners commonly involved in obesity management in Peninsular Malaysia from both the public and private healthcare sectors. Practitioners included medical officers or specialists in primary care, internal medicine and bariatric surgery, and allied health practitioners (dieticians, nutritionists, physiotherapists, occupational therapists, sport science officers, exercise physiologists, clinical psychologists, and counsellors). Participants were eligible to participate if they practised in Peninsular Malaysia, are involved in direct patient care, and have worked in healthcare for at least one year. House officers or doctors in internship were excluded, as well as practitioners who intended to retire or leave the service in six months. We restricted the locations to Peninsular Malaysia and excluded East Malaysia (Sabah, Sarawak and Federal Territory of Labuan) as the healthcare access and internet coverage in East Malaysia are more limited than in Peninsular Malaysia, which might influence our data collection and interpretation.

### Data collection procedure

A convenience sampling method was applied. Participants were recruited through Facebook advertisements, professional networks of research team members and snowball sampling. A Facebook page was set up for this study, and an advertisement was placed on it throughout the data collection period, targeting Facebook users from Peninsular Malaysia who work in healthcare and medical services. The research team members contacted their professional networks in Malaysia and asked them to distribute an electronic study flyers to their members. A reminder was sent by the organisations one month after the flyer’s distribution. The flyers were posted on the Facebook groups of various associations every four weeks with permission from the page administrators, including the Malaysian Medical Association, Malaysian Dietetics’ Association, Malaysian Nutritionists Association, Malaysian Primary Care Network, Malaysian Family Medicine Specialist Association and Malaysian Physiotherapy Association. Potential participants clicked on the survey link embedded in the advertisements and flyers and were redirected to the Qualtrics platform website. They answered the eligibility questions, and only eligible participants were able to continue to the explanatory statement and consent page. Participants provided informed consent before completing the survey. A snowballing approach was used where participants were invited to share the survey link with their colleagues who may be eligible and potentially interested after they completed the survey.

The survey was set to prevent multiple submissions from the same participant. Data on participants’ browsers, operating systems and locations were analysed by the survey website to prevent fraudulent responses. Participants were allowed to save their progress and return to the survey up to three months after they first accessed the survey. Responses were recorded as “incomplete” if the survey was not completed after three months or by the last day of data collection period.

### Measurement instruments

The survey consisted of three sections: practitioners’ perceptions, experiences and needs; UMB Fat; and sociodemographic and clinical-related profile (Additional file 1).

#### Perceptions, experiences and needs of healthcare practitioners

Perceptions, experience and needs questions asked about the practitioners’ attitudes towards obesity and patients with obesity (11 questions), their perceptions of Malaysia’s obesity healthcare system (7 questions), their reasons for not discussing weight with patients (3 questions), and their perceived need to improve their capacity to manage obesity (3 questions). These questions had various response formats, including a 5-point Likert scale, multiple-choice answers, and a 10-point scale. Most of the questions for this section were adapted from the Awareness, Care, and Treatment in Obesity Management International (ACTION-IO) survey [6], with four questions added by our research team. The ACTION-IO survey was a 2018 multinational study on the perception, attitudes, and barriers to effective obesity care that investigated and compared the perspectives of patients and health practitioners [6]. Other than ACTION-IO, the ACTION survey was conducted earlier in the US [41] and Canada [42]. We selected the questions from the section on healthcare practitioners relevant to our study objectives to allow a comparison of the perspectives of Malaysian healthcare practitioners with international sites. The questions were mapped to the three domains of the COM-B model to assist in understanding practitioners’ engagement in obesity management (Additional file 2). The questions were discussed between research team members. Several items were dropped so that the survey was not too long, and the response format for two items was revised from a 5-point to a 10-point scale to provide a more nuanced understanding of practitioners’ perspectives.

#### UMB fat questionnaire

The UMB Fat was developed and validated among psychology students in the United States and New Zealand in 2008 [43]. This scale has been used extensively in public [18, 44] and healthcare settings [45–47] and found to have good internal consistency reliability, with Cronbach’s alpha values ranging from 0.73 to 0.92 [18, 44–47]. The questionnaire consists of 20 items, eight of which are negatively worded, with four domains: negative judgement, distance, attraction, and equal rights [43]. Each domain consists of five items measured on a 7-point Likert scale (“strongly agree” to “strongly disagree”) [43]. In previous studies, the score was usually reported as an average summary score for all items ranging from one to seven, with a higher score indicating a higher level of weight stigma [18, 44–46].

Despite its extensive use, the UMB Fat scale has never been used in Asian countries, particularly Malaysian population. Using the survey data, we conducted a validation study of the UMB Fat questionnaire using Rasch analysis [Authors, 2023, submitted for publication]. The result showed that Rasch analysis supported reporting of the UMB Fat domain scores but not the summary score. Therefore, the level of weight stigma in this survey was measured by the average score for the four UMB Fat domains, and the average summary score for comparison with previous international studies, noting that our Rasch analysis did not support the unidimensionality of the overall score [Authors, 2023, submitted for publication].

#### Sociodemographic and clinical-related profile

We collected sociodemographic variables: age, gender, ethnicity, body mass index category and history of personal weight loss. Clinical-related variables were healthcare profession, work location, healthcare setting, highest academic qualification, duration of service, involvement in obesity management, expertise in obesity management and advanced training in obesity management.

#### Pilot test

The survey was administered in English as the sentences used were simple English and standard medical terms that our target population (healthcare practitioners in Peninsular Malaysia) would likely understand, as most medical and health science degrees in Malaysia are taught in English. We piloted the survey on 23 healthcare practitioners (doctors in primary care and dietitians) to test for face validity, the administration process and data entry preparation. The time taken to complete the survey was between 10 and 18 min. The survey was easily understood by the pilot participants. Based on the responses, we revised the question “What are the top 5 reasons for which you might NOT discuss obesity with a patient?” to “What are your top 5 barriers to obesity management?” for participants involved in obesity clinics.

### Statistical analysis

Data analysis was conducted using SPSS software version 24. Descriptive statistics were used to summarise participants’ characteristics and their perceptions, experiences and needs in managing obesity. Data were reported as frequency, percentage, mean (SD) or median (IQR), as appropriate. A significant *p*-value was set at 0.05, with a 95% confidence interval.

The level of weight stigma measured using the UMB Fat scale was reported as the mean (SD) or median (IQR) of the average summary score (for 20 items) and the average score for each domain (for five items), depending on the distribution of the data. The average score was calculated as the total score for all items divided by the number of items.

We performed multiple linear regressions to determine the factors associated with higher negative judgement towards patients with obesity (domain 1 of the UMB Fat). The independent variables were age, sex, body mass index (BMI), type of profession, health sector, length of service, involvement in an obesity clinic, expertise in obesity management, advanced training in obesity management, the belief that obesity is a chronic disease, degree of comfort in discussing obesity, and previous weight loss success. A two-step modelling approach was adopted, starting with univariate linear regression analysis for each independent variable [48]. Variables with a moderate association (*p* ≤ 0.1) with the outcome of negative judgement were included in the multivariate regression analysis (standard regression model) and retained if *p* ≤ 0.05. Prior to the analysis, preliminary screening of the residual plots were undertaken to ensure that the assumptions of regression analysis (i.e. normality, linearity, homoscedasticity and non-independence of errors) were met. The level of tolerance and variation inflation factor was also inspected to ensure that independent variables were not highly correlated.

### Sample size calculation

The sample size was calculated based on the multiple linear regression analyses undertaken to determine the factors associated with higher negative judgements about patients with obesity using the general formula, n = 50 + 8* m* (where *m* is the number of the independent variables) for testing the multiple correlations [49]. Given that 12 predictors were included in the multivariate regression model (age, sex, BMI, type of profession, health sector, length of service, involvement in an obesity clinic, expertise in obesity management, advanced training in obesity management, the belief that obesity is a chronic disease, degree of comfort in discussing obesity, and previous weight loss success), our sample exceeded the minimum number of participants required for the regression analysis (50 + (8 × 12) = 146).

## Results

**Fig. 1:**
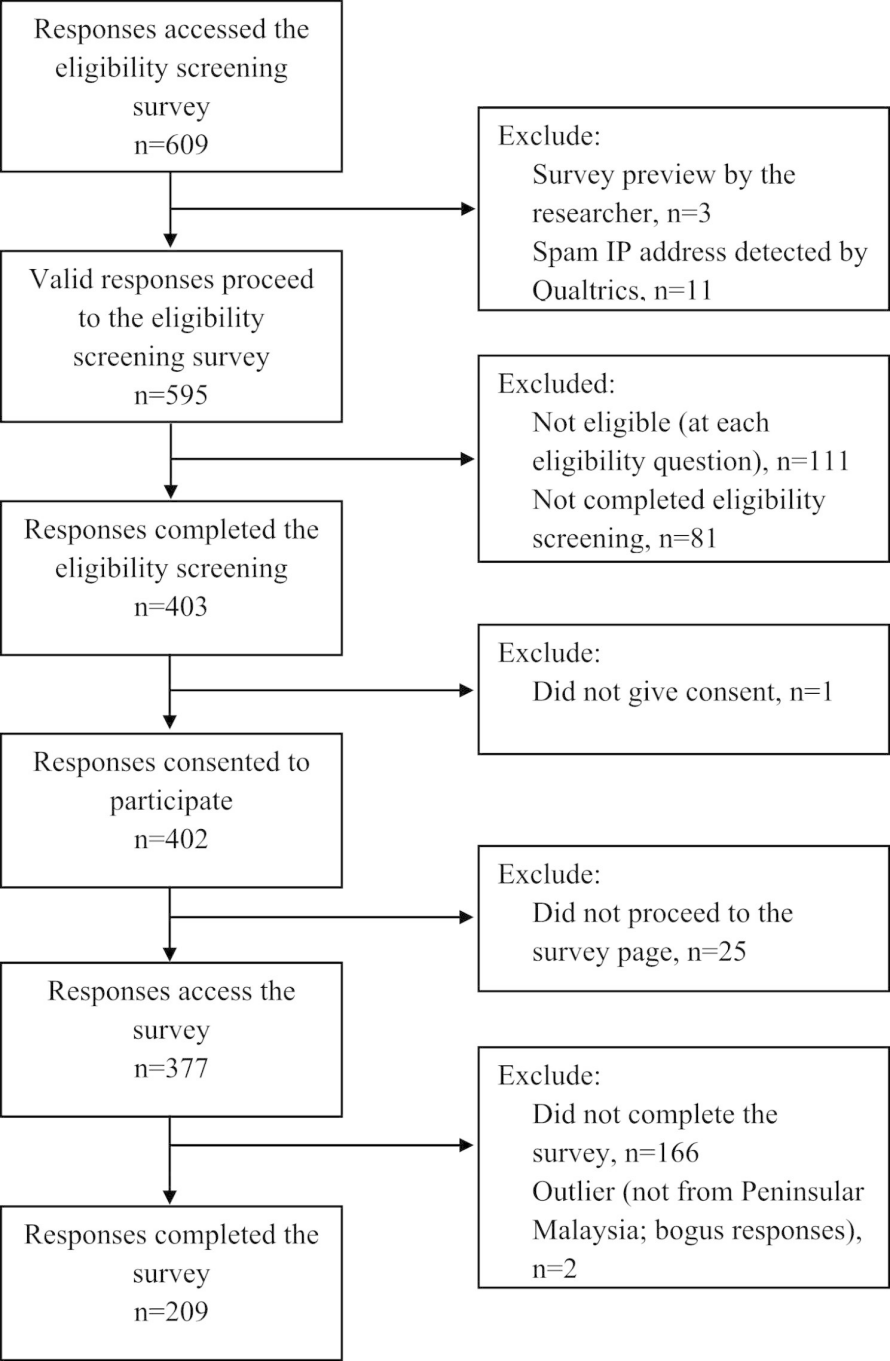
Study flow chart

The survey website received 595 visitors, of which 377 were eligible and agreed to participate, giving a participation rate of 63.4% (399/595) [50]. The survey was completed by 209 participants, with a completion rate of 55.4% (209/377) (Fig. 1) [50].

### Demographic and service characteristics

Data were missing for three items in this section (1.44% of missing data). Table 1 presents the demographic characteristics of the participants. The mean (SD) age was 41.1 (9.5) years. The majority of participants were female (n = 141, 67.5%), of Malay ethnicity (n = 148, 70.8%), and reported having a normal BMI (n = 112, 53.6%). Most worked in the public sector (n = 152, 72.7%) and primary care settings (n = 173, 82.8%). Only 8.6% (n = 18) of the participants were allied health practitioners. The mean (SD) duration of service was 11.9 (8.9) years. Most participants did not work in an obesity clinic (n = 124, 59.3%), did not consider themselves obesity experts (n = 168, 80.4%), and did not have advanced training in obesity management (n = 168, 80.4%).

**Table 1:** Sociodemographic characteristics of the participants, n = 209

Demographic characteristics	All participants (n = 209)	
Gender		
- Male	64	(30.6)
- Female	141	(67.5)
- Prefer to self-identify	1	(0.5)
- Prefer not to answer	3	(1.4)
Ethnicity		
- Malay	148	(70.8)
- Chinese	28	(13.4)
- Indian	24	(11.5)
- Others – Siamese, Dusun	2	(1.0)
- Prefer not to answer	7	(3.3)
Work location		
- Northern region (Perlis, Kedah, P.Pinang, Perak)	41	(19.6)
- Central region (Kuala Lumpur, Putrajaya, Selangor)	62	(29.7)
- Southern region (N.Sembilan, Melaka, Johor)	25	(12.0)
- Eastern region (Pahang, Kelantan, Terengganu)	80	(38.3)
- Missing	1	(0.4)
Healthcare sector		
- Public	152	(72.7)
- Private	45	(21.5)
- Both	12	(5.7)
Health disciplines		
- Primary care	173	(82.8)
- Internal medicine	13	(6.2)
- Bariatric surgery	2	(1.0)
- Allied health	18	(8.6)
- Others	3	(1.4)
Days of direct patient care per week		
- Less than 1 day	5	(2.4)
- At least 1, but no more than 2 days	24	(11.5)
- 3 days or more	177	(84.7)
- Others: twice per month; 11 sessions per week; 1 to 3 days per week	3	(1.4)
Expert in obesity management		
- Yes	26	(12.4)
- No	168	(80.4)
- Prefer not to answer	15	(7.2)
Advanced training in obesity management		
- Yes	35	(16.7)
- No	168	(80.4)
- Prefer not to answer	6	(2.9)
BMI category		
- Underweight	3	(1.4)
- Normal	112	(53.6)
- Overweight	68	(32.5)
- Obese	13	(6.2)
- Prefer not to answer	13	(6.2)
Maintained weight loss		
- Yes	102	(48.8)
- No	92	(44.0)
- Prefer not to answer	15	(7.2)
Involvement in obesity management service		
- Yes	74	(35.4)
- No	124	(59.3)
- Prefer not to answer	11	(5.3)

All data reported as n(%) unless stated otherwise

### Perceptions, experiences and needs in obesity management

**Fig. 2:**
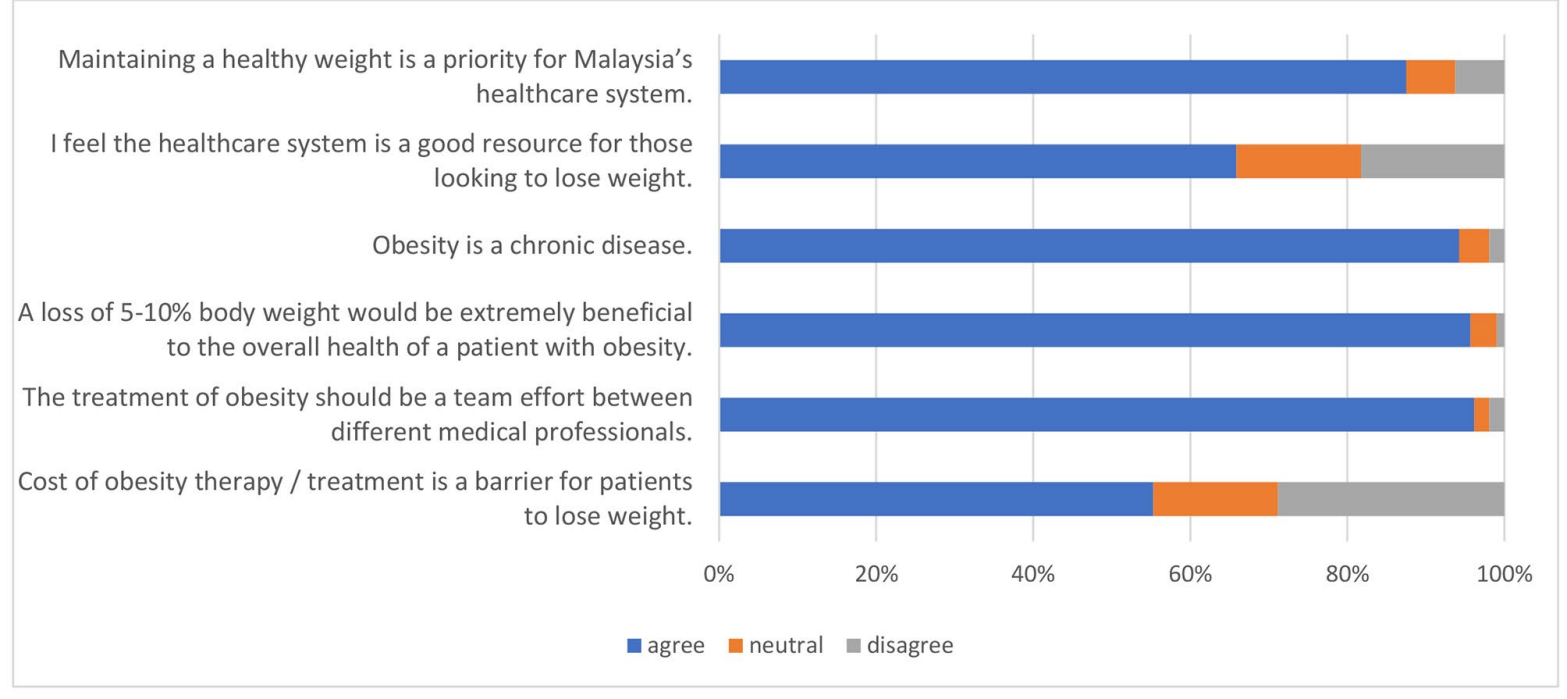
Percentage of participants’ agreement with the statements about their perceptions of the obesity healthcare system, n = 208

Data for two items were missing from this section resulting in less than 1% of missing data. Most participants agreed (answered “agree” and “strongly agree”) with statements that obesity is a chronic disease (n = 196/208, 94.3%) and should be a priority in healthcare (n = 182/208, 87.5%). The majority (n = 200/208, 96.2%) also agreed that obesity management should be multi-disciplinary care between different healthcare professions. However, as shown in Fig. 2, only 65.7% (n = 137/208) of participants agreed that the healthcare system is a good resource for patients with obesity and perceived that the current healthcare system might not be meeting the need of patients with obesity, with a median (IQR) score of 4.00 (3) out of 10.

**Fig. 3:**
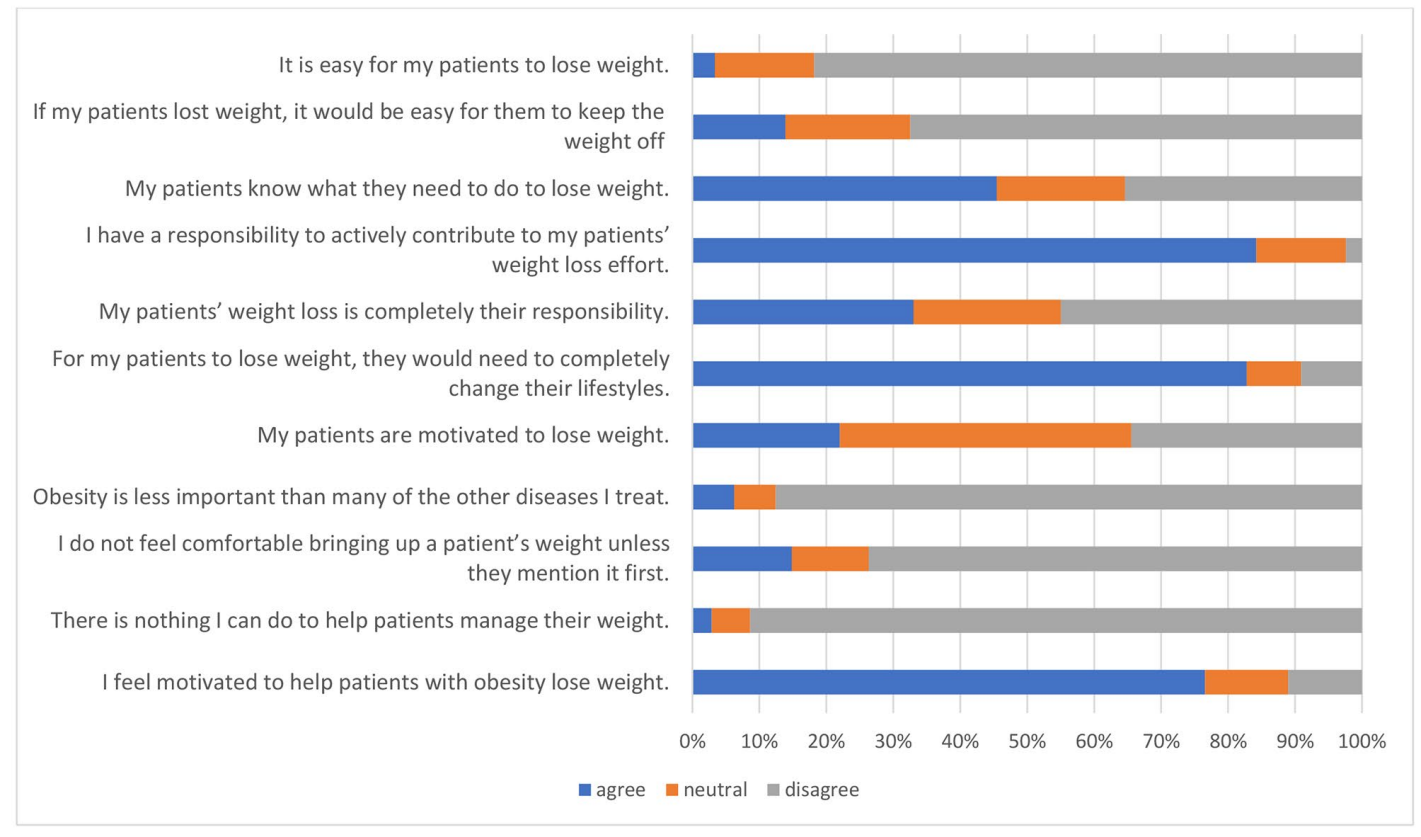
Percentage of participants’ agreement with the statements about their attitudes towards patients with obesity, n = 209

Regarding their responsibility and motivation, most participants (n = 176/209, 84.2%) agreed that they had a responsibility and were motivated to help patients to lose weight (n = 160/209, 76.6%) (Fig. 3). They were also comfortable discussing weight with patients, based on the median (IQR) score of 8.00 (2), the range score of 1 to 10. However, 33% (n = 69/209) of participants placed the responsibility for weight loss on patients, and only 22% (n = 46/209) thought their patients were motivated to lose weight.

**Fig. 4:**
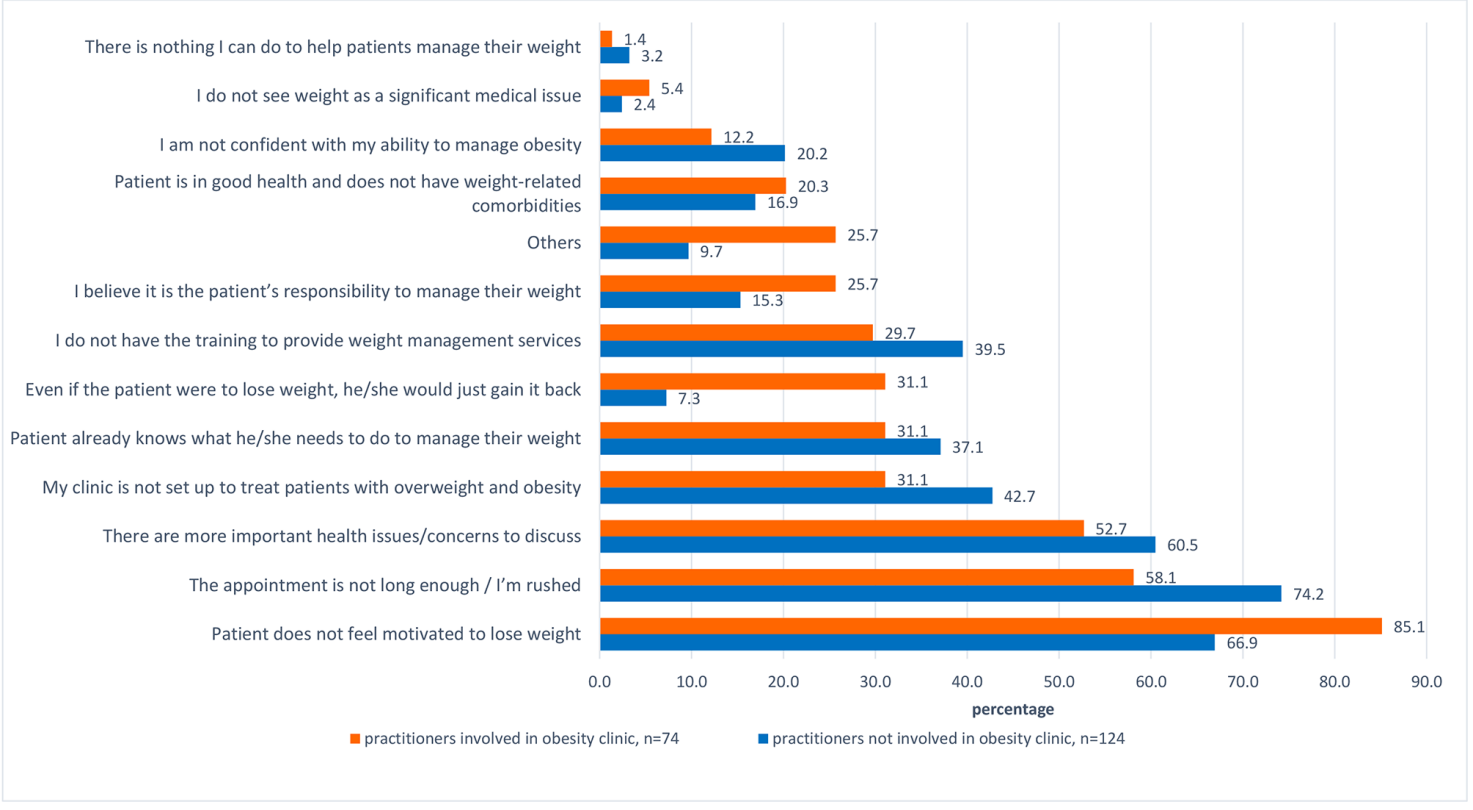
Percentage of reasons for not discussing obesity with patients reported by participants who directly involved and not directly involved in obesity clinic

Figure 4 summarises the participants’ reasons for not discussing obesity with patients. The three most common reasons were not having enough time, patients’ lack of motivation, and having other more important concerns to discuss and these were the same for participants regardless of whether they worked in an obesity clinic or not. Patients’ lack of motivation was the most common reason mentioned by participants directly involved in obesity clinics.

Almost 90% (n = 186/209) of the participants reported needing more support in obesity management. The most common type of support selected by participants who needed more support was accessibility to multi-disciplinary obesity services (n = 169/186, 90.9%) and training in obesity management (n = 165/186, 88.7%). Their preferred training approaches were workshops, certified courses, educational aids, and face-to-face delivery. Apart from training, participants also needed a budget to run obesity management programs at their clinics (n = 145/186, 78%) and guidelines on obesity management (n = 144/186, 77.4%). Sixty-six per cent of participants (n = 123/186) reported needing obesity medication to be made available at health clinics. Of those who reported not needing more support, some already had the support they needed (n = 10/209, 4.8%) and others had alternative priorities (n = 8/209, 3.8%). A small number of participants (n = 4/209, 1.9%) were not interested in obesity management.

### Level of weight stigma and factors associated with negative judgment

The mean (SD) average total UMB-Fat score for all participants was 2.99 (0.87). The scores for each of the four domains are presented in Table 2. Three independent variables (direct involvement in obesity management, having advanced training in obesity and degree of comfort with obesity discussion) were moderately associated with negative judgment in the univariate linear regression analysis, included in the multivariate model (Table 3). However, none of these factors were significantly associated with negative judgements of people with obesity in the final multivariate model. This multivariate regression model explained only 3.1% of the variance in the dependent variable (negative judgment towards patients with obesity). The complete results of the linear regression analysis are presented in Additional file 3.

**Table 2:** The total and average scores for the UMB Fat and each domain, n = 209

Variables (n = 209)	Mean	SD	95% CI
UMB Fat			
Total score (range 20–140) Average score (range 1–7)	59.99 2.99	17.42 0.87	57.61, 62.37 2.88, 3.11
Domain 1: negative judgment			
Total score (range 5–35) Average score (range 1–7)	13.36 2.67	6.17 1.23	12.52, 14.20 2.50, 2.84
Domain 2: distance			
Total score (range 5–35) Average score (range 1–7)	13.78 2.76	5.35 1.07	13.05, 14.51 2.61, 2.90
Domain 3: attraction			
Total score (range 5–35) Average score (range 1–7)	21.78 4.36	5.31 1.06	21.06, 22.51 4.21, 4.50
Domain 4: equal rights			
Total score (range 5–35) Average score (range 1–7)	11.06 2.21	7.23 1.45	10.08, 12.05 2.02, 2.41

**Table 3:** The associated factors for negative judgment from multiple linear regression analysis

Independent variables	B (standardised coefficient) ^a^	Standard Error	*p*-value	95% CI
Degree of comfort with obesity discussions	-0.094	0.050	0.207	-0.163, 0.035
Direct involvement in obesity management	0.092	0.191	0.221	-0.142, 0.611
Advanced training in obesity	0.069	0.250	0.371	-0.269, 0.718

^a^standard multiple regression model was applied

## Discussion

Our study found that health practitioners in this study acknowledged that obesity is a chronic disease and that supporting patients to achieve a healthy weight target should be a priority in healthcare. While practitioners reported having the motivation and capability to discuss weight with patients, they were less likely to discuss obesity with patients due to perceptions that patients lack motivation to lose weight, short consultation times and having other health concerns to handle during consultations. In addition, the practitioners in this study reported that the current healthcare system in Peninsular Malaysia is unsupportive of patients with obesity and practitioners. They need more support to access multi-disciplinary obesity care for their patients and advanced training on obesity management to improve their obesity care delivery. Moderate weight stigma was present among practitioners in our study, but we did not discover any factors associated with negative judgment towards patients with obesity. Weight stigma in healthcare settings in Malaysia needs to be addressed as it is likely to hinder weight discussions with patients as it does in other parts of the world.

The COM-B model provides a framework for understanding how health practitioners engage in obesity care. Practitioners’ beliefs of the severity and importance of obesity, plus their perceived responsibility indicating their reflective motivation for engaging in obesity management [9]. Practitioners in this study reported having the capability for obesity care, based on their high degree of comfort with weight discussion, including initiating the discussion. However, the practitioners’ motivation was limited by social opportunity - their negative perceptions of patients’ lack of interest and motivation to lose weight and the systematic limitations within the healthcare environment, also impacted by the many health issues that need to be managed within a short consultation time.

The perceptions on obesity and barriers to obesity management for our participants are consistent with the findings from the ACTION studies. Obesity used to be considered a risk factor for cardiovascular and metabolic diseases [1] and is now believed to be a chronic disease by our survey participants. This shift in belief is also reported in previous ACTION studies [6, 41, 42, 51], which is in line with the international recognition of obesity as a chronic disease by the World Obesity Federation [52], the European Association for the Study of Obesity [53], The Obesity Society [54] and the American Medical Association [55]. The three most common reasons for not discussing weight reported in our survey also have striking similarities with the earlier ACTION studies [6, 42, 56]. It appears that practitioners across the globe, from western and Asian, higher and lower income countries, are all reporting the same barriers to providing high-quality obesity care. One of the main barriers to obesity management reported by practitioners in our study and others was patients’ low motivation and interest [7, 12, 14]. However, contrary evidence shows that practitioners tend to underestimate patients’ motivation to lose weight [14, 57]. In contrast, patients were concerned about their excessive weight and had tried to lose weight but had limited success [6]. It is possible that only focusing on kilogram loss [57] rather than a more holistic view of health means that practitioners perceive patients to have low motivation when they do not reduce their weight.

Practitioners in our survey reported several limitations of the current healthcare system, most importantly the lack of access to multi-disciplinary care. Public primary care clinics in Malaysia have the potential to develop multi-disciplinary obesity care, given the availability of allied healthcare professionals in the clinics [58] and accessibility to patients with obesity [59]. However, having human resources without advanced training may not be sufficient to improve obesity care. Our survey shows that despite being comfortable discussing weight, practitioners still reported feeling the need for advanced training in obesity management. This finding supports an earlier study in Malaysia reporting that practitioners have moderate self-efficacy in delivering obesity counselling to patients [37]. Obesity training could include the complex process of body weight regulation and awareness of weight stigma in healthcare settings to reduce perceived personal attribution of obesity [24, 60], given the well-known negative influences of weight stigma in healthcare on patients’ health and well-being [23, 32]. The need for an updated Malaysian obesity management guideline is critical, as the last one was published in 2004 [61] and is no longer relevant given recent advances in obesity research and evidence-based clinical management. More financial support for obesity clinics and improved accessibility of obesity medications were also mentioned by practitioners and this may warrant consideration by policymakers in Malaysia.

Our participants’ weight stigma level was in the middle range of the UMB Fat scale, similar to other health practitioners in international studies [46, 47]. The scores for the three domains relevant to healthcare settings, i.e. negative judgment, distance and equal rights domains, were moderate, almost similar to other general population studies [45, 62]. However, the domain scores were not reported in previous studies in healthcare settings [46, 47]. Despite the moderate levels, the scores indicated the presence of weight stigma among Malaysian healthcare practitioners. In addition, practitioners’ negative perceptions of patients’ lack of motivation could potentially be another sign of negative judgment and stigma towards patients with obesity [63], which had been reported as one of the main reasons for not discussing obesity with the patients. Therefore, weight stigma should be appropriately addressed to avoid further impacts on healthcare delivery in our population. Contrariwise to the literature [34], we did not identify any significant factors associated with negative judgment in our study cohort.

### Strengths and limitations

This survey was informed by underlying theories in its development and interpretation, which give strength to the design. We adapted previous questions from established international studies in multiple countries on practitioners’ perceptions on obesity management, the ACTION-IO study, allowing a comparison of Malaysian practitioners’ perceptions with the global views. In addition, the UMB Fat questionnaire used in this survey has been validated for our population. Nevertheless, the survey findings should be interpreted within the limitations of this survey. This was a cross-sectional study where exposure and outcomes were measured at the same time, making it relatively difficult to establish a causal relationship. As the reference population was not accessible to the researchers for probability sampling, a convenient sampling method was applied in this survey. As a non-probability sampling method, convenience sampling has several limitations over probability sampling, including being less representative of the population and the risk of selection bias. However, we have taken several steps to improve our survey’s credibility and be as representative of the target population as possible [64]. The measures include recruiting as many participants as possible during the data collection period and using multiple approaches to recruit participants, including social media, professional networks and adopting a snowballing approach. In addition, the demographic characteristics of our participants match the target population in terms of age group, public and private sector distribution, and geographical distribution between the west and east coasts of Peninsular Malaysia, noting that our responses from female practitioners were slightly higher than the population [65]. The interpretation of the findings should also consider the possibility that practitioners who were not interested in obesity management or had a higher weight stigma might choose not to participate. Besides, our participants contained few allied health professionals and trained obesity experts, meaning that generalisability was limited to those groups.

### Future directions

Obesity is a complex disease that requires a multifactorial approach to improve the effectiveness of obesity management [66]. The combination of the COM-B model and attribution theory strengthens the exploration of Malaysian health practitioners’ perceptions of obesity management. Our findings provide a preliminary understanding of how causal attribution of obesity may influence practitioners’ engagement in obesity management, particularly their opportunity to manage obesity. Considering that the COM-B model is the central behavioural analysis for the Behaviour Change Wheel intervention framework [9], the knowledge of practitioners’ weight stigma and its influence on the COM-B domains could be incorporated into the intervention framework for obesity management in Malaysia to make it more comprehensive. Nevertheless, validation studies are needed to accurately measure the constructs of capability, opportunity, motivation, and causal beliefs in this area and their contribution to practitioners’ engagement in obesity management.

## Conclusion

This study highlighted the perspectives of healthcare practitioners on obesity management in Peninsular Malaysia. Obesity was recognised by health practitioners in this study as a chronic disease and an important healthcare priority. While practitioners reported that they had the motivation and capability to discuss obesity with their patients, their opportunity to engage in obesity discussions was limited by the systematic issues within the current healthcare system and the social opportunity of perceiving patients’ low motivation to lose weight. The summary UMB Fat score and the individual scores for each domain indicated moderate stigma among our participants. Despite this, practitioners’ negative perceptions of patients’ lack of motivation may indicate stigma towards patients with obesity that potentially influences engagement with obesity discussions. Nonetheless, no significant personal and clinical-related factors were associated with negative judgment of patients with obesity in our study participants. Practitioners needed more systematic support, particularly with availability and accessibility of multi-disciplinary obesity care and advanced training in obesity management and stigma awareness.

## Electronic supplementary material

Below is the link to the electronic supplementary material.

Supplementary Material 1

Supplementary Material 2

Supplementary Material 3

## Data Availability

All available data are presented in the article and its Supporting Information files. The datasets generated and/or analysed during the current study are not publicly available due ethical reason as it is not covered by the informed consent provided by the study participants.

## Abbreviations

ACTION-IO: Awareness, Care, and Treatment in Obesity Management International
BMI: body mass index
COM-B: Capability, Opportunity, Motivation and Behaviour
STROBE: STrengthening the Reporting of OBservational studies in Epidemiology
UMB Fat: Universal Measures of Bias – Fat
