# Supplementary material for: The perceptions of healthcare practitioners on obesity management in Peninsular Malaysia: a cross-sectional survey
Source: BMC Health Serv Res. 2023 Jul 10;23:744. doi: 10.1186/s12913-023-09759-z (PMC10334633; doi:10.1186/s12913-023-09759-z)
Supplement: Supplementary file 1 — Supplementary Material 1 [file 12913_2023_9759_MOESM1_ESM.pdf]

Intro

PROVIDERS' PERCEPTIONS, EXPERIENCES AND NEEDS IN MANAGING OBESITY IN PENINSULAR MALAYSIA

We appreciate your interest in participating in this survey.

This study aims to explore healthcare providers' perspectives of healthcare for obesity in Peninsular Malaysia. The questions are about your perceptions of patients with obesity and the healthcare system for obesity management, your experience in managing patients with obesity and your demographic and work profile. All of your answers are confidential and anonymous.

UMB Fat

Q1. Please answer all of the following questions using this scale.

	Please choose one answer from the scale 1 to 7 belo					
	1 - Strongly agree	2 - Moderately agree	3 - Slightly agree	4 - Neither agree nor disagree	5 - Slightly disagree	6 - Moderatel disagree
1. People with obesity tend toward bad behaviour	○	○	○	○	○	○
2. People with obesity are sloppy	○	○	○	○	○	○
3. Sometimes I think that people with obesity are dishonest	○	○	○	○	○	○
4.People with obesity have bad hygiene	○	○	○	○	○	○
5. In general, people with obesity don’t think about the needs of other people	○	○	○	○	○	○
6. I would not want to have a person with obesity as a roommate	○	○	○	○	○	○

	Please choose one answer from the scale 1 to 7 belo					
	1 - Strongly agree	2 - Moderately agree	3 - Slightly agree	4 - Neither agree nor disagree	5 - Slightly disagree	6 - Moderatel disagree
7. I like people with obesity	○	○	○	○	○	○
8. I don’t enjoy having a conversation with a person with obesity	○	○	○	○	○	○
9. I would be comfortable having a person with obesity in my group of friends	○	○	○	○	○	○
10. I would like having a person with obesity at my place of worship or community centre	○	○	○	○	○	○
11. I find people with obesity attractive	○	○	○	○	○	○
12. People with obesity make good romantic partner	○	○	○	○	○	○
13. I find people with obesity to be sexy	○	○	○	○	○	○
14. People with obesity are a turn-off	○	○	○	○	○	○
15. I find people with obesity pleasant to look at	○	○	○	○	○	○
16. Special effort should be taken to make sure that people with obesity have the same rights and privileges as other people	○	○	○	○	○	○
17. Special effort should be taken to make sure that people with obesity have the same salaries as other people	○	○	○	○	○	○
18. Special effort should be taken to make sure that people with obesity have the same educational opportunities as other people	○	○	○	○	○	○
19. Special effort should be taken to make sure that people with obesity have the same housing opportunities as other people	○	○	○	○	○	○
20. I try to understand the perspective of people with obesity	○	○	○	○	○	○

attitudes

Q2. Thinking of your patients with obesity as a whole, please indicate how much you agree with each of the following.

Use a scale where 1 means "Do not agree at all" and 5 means "Completely agree"

	Please choose one answer from the scale				
	1 - Do not agree at all	2	3	4	5 - Completely agree
1. It is easy for my patients to lose weight.	○	○	○	○	○
2. If my patients lost weight, it would be easy for them to keep the weight off (maintain the new weight).	○	○	○	○	○
3. My patients know what they need to do to lose weight.	○	○	○	○	○
4. I have a responsibility to actively contribute to my patients' weight loss effort.	○	○	○	○	○
5. My patients' weight loss is completely their responsibility.	○	○	○	○	○
6. For my patients to lose weight, they would need to completely change their lifestyles.	○	○	○	○	○
7. My patients are motivated to lose weight.	○	○	○	○	○
8. Obesity is less important than many of the other diseases I treat.	○	○	○	○	○
9. I do not feel comfortable bringing up a patient's weight unless they mention it first.	○	○	○	○	○
10. There is nothing I can do to help patients manage their weight.	○	○	○	○	○
11. I feel motivated to help patients with obesity lose weight.	○	○	○	○	○

RQ 2 (perceptions on system)

Q3. Please indicate how much you agree with the following statements regarding obesity and weight management.

Use a scale where 1 means "Do not agree at all" and 5 means "Completely agree"

	Please choose one answer from the scale				
	1 - Do not agree at all	2	3	4	5 - Completely agree
1. Maintaining a healthy weight is a priority for Malaysia's healthcare system.	○	○	○	○	○
2. I feel the healthcare system (clinics, hospitals, allied health services etc.) is a good resource for those looking to lose weight.	○	○	○	○	○
3. Obesity is a chronic disease.	○	○	○	○	○
4. A loss of 5-10% body weight would be extremely beneficial to the overall health of a patient with obesity.	○	○	○	○	○
5. The treatment of obesity should be a team effort between different medical professionals.	○	○	○	○	○
6. Cost of obesity service and treatment is a barrier for patients to lose weight.	○	○	○	○	○

Q4. To what extent is the Malaysian healthcare system currently meeting the needs of patients with obesity?

Please use a scale where 1 means our healthcare system is "Not at all meeting the needs" and 10 means "Completely meeting the needs".

1 2 3 4 5 6 7 8 9 10

RQ 3 - experience

Q5. Do you currently work in an obesity management clinic/service?

- ○ Yes
- ○ No
- ○ Prefer not to answer

Q6. How comfortable are you in having discussions with your patients about their weight?

Please use a scale where 1 means you are "Not at all comfortable" and 10 means you are "Extremely comfortable"

1 2 3 4 5 6 7 8 9 10

Q7a. What are the top 5 reasons for which you might **NOT** discuss obesity with a patient?

Please select up to 5 (FIVE) items only

- ☐ 1. The appointment is not long enough / I'm rushed
- ☐ 2. There are more important health issues/concerns to discuss
- ☐ 3. My clinic is not set up to treat patients with overweight and obesity
- ☐ 4. I am not confident with my ability to manage obesity
- ☐ 5. I do not see weight as a significant medical issue
- ☐ 6. Patient is in good health and does not have weight-related comorbidities
- ☐ 7. I believe it is the patient's responsibility to manage their weight
- ☐ 8. Patient does not feel motivated to lose weight
- ☐ 9. Patient already knows what he/she needs to do to manage their weight
- ☐ 10. Even if the patient were to lose weight, he/she would just gain it back
- ☐ 11. There is nothing I can do to help patients managing their weight
- ☐ 12. I do not have the training to provide weight management services
- ☐ 13. Others (please specify) __________

Q7b. What are your top 5 barriers to obesity management?

Please select up to 5 (FIVE) items only

- ☐ 1. The appointment is not long enough / I'm rushed
- ☐ 2. There are more important health issues/concerns to discuss
- ☐ 3. My clinic is not set up to treat patients with overweight and obesity
- ☐ 4. I am not confident with my ability to manage obesity
- ☐ 5. I do not see weight as a significant medical issue
- ☐ 6. Patient is in good health and does not have weight-related comorbidities
- ☐ 7. I believe it is the patient's responsibility to manage their weight
- ☐ 8. Patient does not feel motivated to lose weight
- ☐ 9. Patient already knows what he/she needs to do to manage their weight
- ☐ 10. Even if the patient were to lose weight, he/she would just gain it back

☐ 11. There is nothing I can do to help patients managing their weight

☐ 12. I do not have the training to provide weight management services

☐ 13. Others (please specify) ________

RQ 4 - HCP Needs

Q8. Would you like more support to assist you to manage patients with obesity?

○ Yes

○ No, I am not interested

○ No, I have other priorities at the moment

○ No, I already have enough support

○ No, I have other reasons (please specify) ________

Q9. What types of support do you think would assist you to manage patients with obesity?

Please select ALL that apply.

☐ 1. Training in obesity management

☐ 2. Additional staff in your clinic (please specify the category of staff)
____

☐ 3. Make obesity medication available at health clinics

☐ 4. Budget for the clinic to run obesity management programs

☐ 5. Obesity management guidelines

☐ 6. Flow of management for patients with obesity

☐ 7. Increased availability and accessibility to multidisciplinary obesity services

☐ 8. Others (please specify) ________

Q10. How would you prefer the obesity management training to be delivered?

Please select all that apply.

☐ 1. Talk or lecture (e.g 1 hour of continuous medical education - CME)

☐ 2. Workshop (e.g 1 to 2 days workshop)

- ☐ 3. Certified courses (e.g SCOPE certification)
- ☐ 4. Educational aid (e.g flip chart, video etc)
- ☐ 5. Online training
- ☐ 6. Face-to-face training
- ☐ 7. Free training
- ☐ 8. Willing to pay for obesity training
- ☐ 9. Others (please specify) ____________

demography and service

Thank you for your responses.
We would like to gather some basic information about yourself and your service.

1. What is your age (in years)?

____

2. What is your gender?

- ○ Male
- ○ Female
- ○ Prefer to self-identify ____________
- ○ Prefer not to answer

3. What is your ethnicity?

- ○ Malay
- ○ Chinese
- ○ Indian
- ○ Others, please specify: ____________
- ○ Prefer not to answer

4. Where do you work? (please write the district and state of your health facility)

District

State

5. In which healthcare sector do you work?

- Public sector
- Private sector
- Both public and private
- Others, please specify:

6. What is your profession?

- Internal medicine specialist (including any subspecialties in internal medicine)
- Family medicine specialist
- Bariatric surgeon
- Medical officer in internal medicine
- Medical officer in family medicine
- Medical officer in bariatric surgery
- Dietitian
- Nutritionist
- Physiotherapist
- Occupational therapist
- Sport science officer
- Exercise physiologist
- Clinical psychologist
- Counsellor
- Others, please specify:

7. How long have you been working as a healthcare provider in this discipline? (in months and/or years)

Months

Years

8. How many days are you involved in direct patient care per week?

- Less than 1 day per week
- At least 1, but no more than 2 days per week
- 3 days or more per week
- Others, please specify:

9. Do you consider yourself to be an expert in obesity management?

- Yes
- No
- Prefer not to answer

10. Do you receive any advanced training in obesity management beyond undergraduate training?

- Yes
- No
- Prefer not to answer

11. What is your self-reported body mass index (BMI) category?

- Underweight
- Normal
- Overweight
- Obese
- Prefer not to answer

12. Have you ever maintained significant weight loss? (e.g lost 5-10% of your weight and maintain it for more than 12 months)

- Yes
- No
- Prefer not to answer

Powered by Qualtrics
