# Supplementary material for: The perceptions of healthcare practitioners on obesity management in Peninsular Malaysia: a cross-sectional survey
Source: BMC Health Serv Res. 2023 Jul 10;23:744. doi: 10.1186/s12913-023-09759-z (PMC10334633; doi:10.1186/s12913-023-09759-z)
Supplement: Supplementary file 2 — Supplementary Material 2 [file 12913_2023_9759_MOESM2_ESM.docx]

Mapping of the survey items according to the domains of the COM-B model

| The domain of the COM-B model |  | items in questionnaire |
| --- | --- | --- |
| motivation - reflective | patients | Q2.1. It is easy for my patients to lose weight |
| motivation - reflective |  | Q2.2. If my patients lost weight, it would be easy for them to keep the weight off (maintain the new weight). |
| opportunity - social |  | Q2.3. My patients know what they need to do to lose weight. |
| opportunity - social |  | Q2.5. My patients’ weight loss is completely their responsibility. |
| opportunity - social |  | Q2.6. For my patients to lose weight, they would need to completely change their lifestyles. |
| motivation - reflective |  | Q2.7. My patients are motivated to lose weight. |
| motivation - reflective | HCP | Q2.4. I have a responsibility to actively contribute to my patients’ weight loss effort. |
| motivation - reflective |  | Q2.8. Obesity is less important than many of the other diseases I treat. |
| capability - psychological and physical |  | Q2.9. I do not feel comfortable bringing up a patient’s weight unless they mention it first. |
| motivation - reflective |  | Q2.10. There is nothing I can do to help patients manage their weight. |
| motivation - reflective |  | Q2.11. I feel motivated to help patients with obesity lose weight. |
|  |  |  |
| capability - psychological and physical |  | Q6. How comfortable are you in having discussions with your patients about their weight? |
|  |  |  |
| opportunity - physical | system | Q3.1. Maintaining a healthy weight is a priority for Malaysia’s healthcare system. |
| opportunity - physical |  | Q3.2. I feel the healthcare system (clinics, hospitals, allied health services etc.) is a good resource for those looking to lose weight. |
| motivation - reflective |  | Q3.3. Obesity is a chronic disease. |
| capability - psychological |  | Q3.4. A loss of 5-10% body weight would be extremely beneficial to the overall health of a patient with obesity. |
| opportunity - physical |  | Q3.5. The treatment of obesity should be a team effort between different medical professionals. |
| opportunity - physical |  | Q3.6. Cost of obesity service and treatment is a barrier for patients to lose weight. |
|  |  |  |
| opportunity - physical |  | Q4. To what extent is the Malaysian healthcare system currently meeting the needs of patients with obesity? |
|  |  |  |
|  | barriers | Q7a/Q7b. What are the top 5 reasons for which you might NOT discuss obesity with a patient? |
| opportunity - physical |  | 1. The appointment is not long enough / I’m rushed |
| motivation - reflective |  | 2. There are more important health issues/concerns to discuss |
| opportunity - physical |  | 3. My clinic is not set up to treat patients with overweight and obesity |
| capability - psychological and physical |  | 4. I am not confident with my ability to manage obesity |
| motivation - reflective |  | 5. I do not see weight as a significant medical issue |
| motivation - reflective |  | 6. Patient is in good health and does not have weight-related comorbidities |
| motivation - reflective |  | 7. I believe it is the patient’s responsibility to manage their weight |
| opportunity - social |  | 8. Patient does not feel motivated to lose weight |
| opportunity - social |  | 9. Patient already knows what he/she needs to do to manage their weight |
| motivation - reflective |  | 10. Even if the patient were to lose weight, he/she would just gain it back |
| motivation - reflective |  | 11. There is nothing I can do to help patients managing their weight |
| capability - psychological and physical |  | 12. I do not have the training to provide weight management services |
|  |  | 13. Others (please specify) |
