# Supplementary material for: The perceptions of healthcare practitioners on obesity management in Peninsular Malaysia: a cross-sectional survey
Source: BMC Health Serv Res. 2023 Jul 10;23:744. doi: 10.1186/s12913-023-09759-z (PMC10334633; doi:10.1186/s12913-023-09759-z)
Supplement: Supplementary file 3 — Supplementary Material 3 [file 12913_2023_9759_MOESM3_ESM.docx]

Additional file 3: Multiple linear regression analysis

Table 1: Results for simple linear regression analysis

| Dependent variable | Independent variables | R2 | Adjusted R2 | B (unstandardized coefficient) | Standard error | *p*-value | 95% CI |
| --- | --- | --- | --- | --- | --- | --- | --- |
| Negative judgment | Age | 0.000 | - 0.005 | -0.001 | 0.009 | 0.938 | -0.019, 0.017 |
|  | Duration of service | 0.000 | - 0.005 | - 0.002 | 0.010 | 0.847 | -0.021, 0.017 |
|  | Degree of comfort with obesity discussions | 0.015 | 0.010 | - 0.082 | 0.047 | 0.082* | -0.175, 0.010 |
|  | Sex | 0.007 | 0.003 | - 0.229 | 0.186 | 0.219 | -0.596, 0.137 |
|  | Healthcare sector | 0.001 | -.0.004 | - 0.099 | 0.192 | 0.609 | -0.477, 0.280 |
|  | Profession | 0.002 | - 0.003 | - 0.200 | 0.305 | 0.512 | -0.801, 0.401 |
|  | BMI underweight | 0.002 | - 0.003 | - 0.426 | 0.720 | 0.555 | -1.845, 0.994 |
|  | BMI normal | 0.004 | - 0.001 | - 0.165 | 0.178 | 0.357 | -0.516, 0.187 |
|  | Previous success with weight loss | 0.006 | 0.001 | -0.197 | 0.177 | 0.268 | -0.547, 0.153 |
|  | Direct involvement in obesity management | 0.015 | 0.010 | 0.313 | 0.180 | 0.084* | -0.043, 0.449 |
|  | Expert in obesity management | 0.001 | - 0.004 | 0.099 | 0.261 | 0.704 | -0.415, 0.614 |
|  | Advanced training in obesity | 0.014 | 0.009 | 0.383 | 0.228 | 0.095* | -0.068, 0.833 |
|  | Belief about obesity is a chronic disease | 0.001 | - 0.004 | - 0.136 | 0.368 | 0.711 | -0.371, 0.711 |

*Independent variables with p-value <0.1 are selected for multivariate analysis

Table 2: Results for multiple linear regression analysis

| Independent variables | B (standardised coefficient) ^a^ | Standard Error | P value | 95% CI |
| --- | --- | --- | --- | --- |
| Degree of comfort with obesity discussions | -0.094 | 0.050 | 0.207 | -0.163, 0.035 |
| Direct involvement in obesity management | 0.092 | 0.191 | 0.221 | -0.142, 0.611 |
| Advanced training in obesity | 0.069 | 0.250 | 0.371 | -0.269, 0.718 |

^a^ standard multiple regression model was applied.

Checking the assumptions

Multicollinearity was checked and not found. No correlation between independent variables above 0.9, a tolerance value of less than .10, or a VIF value of above 10 which indicated multicollinearity.

Normality check using the Normal Probability Plot (P-P) showed points lay in a reasonably straight diagonal line from the bottom left to the top right which suggested no major deviations from normality.

From the scatterplot, the residuals were roughly rectangularly distributed with a concentrated of scores along the center, with the residuals trailing off roughly symmetrically from the center, supporting normality, linearity and homoscedasticity.

No outlier was detected on the Scatterplot, i.e. cases that have a standardised residual of more than 3.3 or less than –3.3. The Mahalanobis distance value did not exceed the maximum value for 3 independent variables.

Evaluation of the model

The R square was used, and the value was 0.031. This model which includes the degree of comfort with obesity discussions, direct involvement in obesity care, and advanced training in obesity care explains only 3.1% of the variance in the dependent variable (negative judgment towards patients with obesity). The p-value was 0.117 which was not significant.

Evaluating each of the independent variables

The independent variable “comfortability in having obesity discussions with patients” makes the strongest unique contribution to explain the dependent variable. However, the standardised coefficients of all three independent variables were very small (<0.1) and the p-values were >0.05 indicating the variables were not making a significant unique contribution to the prediction of the dependent variable.
